# The social infrastructure of online marketplaces: Trade, work and the interplay of decided and emergent orders

**DOI:** 10.1111/1468-4446.12965

**Published:** 2022-06-30

**Authors:** Patrik Aspers, Asaf Darr

**Affiliations:** ^1^ University of St Gallen St. Gallen Switzerland; ^2^ The University of Haifa Haifa Israel

**Keywords:** economic sociology, Etsy, market design, market work, organization

## Abstract

This study is designed to remedy the tendency of existing studies to analyze online marketplaces as either sites of work or trading arenas. We argue that the theoretical notion of “social infrastructure” is particularly apt to offer a comprehensive framework that captures the unique intersection of work and trade in online marketplaces. We study the social infrastructure of an online marketplace: the institutions, conditions and forms, and the horizontal and vertical ties between actors that organize work and enable trading. The social infrastructure of online marketplaces deserves research attention because it represents an essential condition for economic activities. In our empirical section we focus on the online marketplace Etsy to illustrate our comprehensive theoretical framework and we identify a complex dynamic between the decided and emergent order of the online marketplace. We demonstrate that the attempt to superimpose order through the constitution of an online marketplace is challenged by sellers and buyers. We find that both dimensions, work and trade, provide actors with material and symbolic resources that inform their strategies and economic actions. The article suggests that “social infrastructure” is a concrete theoretical tool for analyzing online marketplaces that complements existing research on platforms and ecosystems.

## 1 INTRODUCTION

Sociological studies of the platform economy (e.g., Van Dijck, et al., 2018) can be divided into two research streams. The largest stream, originating in the sociology of work and labor process theory, treats platforms as sites of work and employment (Schor, et al., 2020; Vallas & Schor, 2020), as the basis of the gig economy (Aloisi, 2016; Cherry & Aloisi, 2017; Flanagan, 2017), a site of emergent forms of workers' control and resistance (Newlands, 2021), or as a major challenge for labor unions and government regulation of work and employment. The second, and much smaller stream of research is mainly economic‐sociological and depicts platforms as trading arenas, employing key theoretical concepts such as market uncertainty (Beckert & Bronk, 2018), trust (Abrahao, et al., 2017) and reputation systems (Diekmann, et al., 2013), a topic also explored by business scholars. Each of these streams provide important insights into distinct facets of digital platforms. In practice, many platforms are both sites of work and employment for workers and small entrepreneurs, and trade arenas, in which sellers and buyers engage to close deals. To fully understand the complex social dynamics of online platforms, a comprehensive theoretical approach is required that can effectively bring together insights from both streams.

In this article we argue that the theoretical notion of “social infrastructure”, originally developed and applied by economic sociologists, is particularly apt to describe the unique intersection of work and trade represented by trading platforms. We apply the notion of “social infrastructure” to the study of a particular type of digital platform—what we conceptualize as an online marketplace. We ask how the social infrastructure comes about that facilitates the trading of crafts and arts, and which also regulates the work of many independent shop‐owners in the major online marketplace Etsy. We define the social infrastructure of online marketplaces as the *socially produced institutions, conditions and forms, and the hierarchical and horizontal ties between actors that enable work and trade.* Research on the economic aspects of platforms is large and growing, but connections to the marketplace literature are not always clear. Different aspects of the platform economy, including legal, political and economic, have been addressed (e.g., Parker, et al., 2016), and a plethora of terms have been used, which has caused conceptual confusion (Spulber, 2019, p. 160). The notion of a social infrastructure of marketplaces is concrete and narrow and it thereby complements broader notions such as “digital platforms” (Schüßler, et al., 2021), “platform economy” (Kenney, et al., 2021), “ecosystems” (Jacobides, et al., 2019), and “platform society” (Van Dijck, et al., 2018).

Social infrastructure has been seen as a central theoretical construct by scholars from different traditions within economic sociology, which was originally applied only to markets or marketplaces. Fligstein argues that infrastructure is key to analyzing economic exchange, because “markets depend on extensive social infrastructure … stable states and currency, the rule of law, functioning property rights, governance structure, and rules of exchange” (2005: 194). MacKenzie (2006, p. 207) and Abolafia (1996, p. 41) have also analyzed social infrastructure as a condition of trade. Insights about conventions, norms, virtues and institutions for trade in marketplaces that support economic exchange appear in the works of other economic sociologists (cf. Preda, 2009, p. 41; Weber, 1978, pp. 319–325), and are also part of what we define as the social infrastructure of markets. Trust is a key institution and a precondition for any exchange (Durkheim [1895] 1984), as are market devices (Callon, et al., 2007), including algorithms which govern trade and traders, and even accounting.

In this article we examine Etsy, a central online marketplace that also constitutes a site of work. “Marketplace” in our study is analytically distinct from the notion of a “market”. Both markets and marketplaces facilitate trade, but a key difference between a marketplace and a market is that markets can, but do not have to be organized. Historically, markets grew out of marketplaces and were characterized by the lack of a physical point of reference (Jevons, 1871, p. 84). By the term “marketplace” we mean *an organized place, whether physical (e.g., a weekly trading event in the town square) or virtual (e.g., an electronic platform, such as Etsy), for trade* (cf. Plattner, 1989, p. 171). Though it is clear to many that marketplaces are organized, few researchers present a detailed account of what “organization” means. Historically, marketplaces were controlled and organized almost exclusively from “above” (Braudel, 1992), at least in Europe. Under the protection of princes, traders could interact in a safe environment and develop ties that fostered mutual trust (Schieffer, 1976, pp. 124–128). Importantly, there are several studies that describe the combination of organized and mutually adjusted order in marketplaces, of which Michèle de la Pradelle's (2006) study of the weekly street market in Carpentras in France is the most detailed and illuminating (see, e.g., Aspers & Darr, 2018; Brunsson & Jutterström, 2018; Dewey, 2020; Geertz, 1979). The idea that grassroots elements of social infrastructure emerge out of social engagement among actors is also supported by studies of sales floors in diverse contexts (Clark & Pinch, 1995; Darr, 2006).

The unique contribution of this article is to show how concrete theoretical tools taken primarily from economic sociology and organization theory, and enriched by notions from the sociology of work and studies of market work, can fruitfully be employed to understand and analyze how social infrastructure comes about in online marketplaces to enable work and trading. The existing literature on trade platforms addresses—sometimes implicitly and sometimes partially—the organization and regulation of platforms (cf. Dolata, 2019, pp. 184–185) and their making of infrastructure (Kornberger, et al., 2017), infrastructure and marketplaces (Frenken & Fuenfschilling, 2020), or ecosystems (e.g., Gawer, 2014) but does not put it together in a more general theoretical framework for online marketplaces that combines work organization and regulation and partial organization of trade, as is done in this article (cf. Pais & Stark, 2020).

## 2 THE SOCIAL INFRASTRUCTURE OF ONLINE MARKETPLACES

Our study is in line with economic sociologists who have studied, directly or indirectly, social infrastructure online (Beunza, 2019; Dobeson, 2019; Kirchner & Schüßler, 2019; MacKenzie & Millo, 2003). Based on this literature we suggest that it is useful, for analytic purposes, to draw a line between decided and emergent social infrastructure. Decided (or organized) social infrastructure results from decisions made by organizers (Ahrne, et al., 2015) who can implement them for the marketplace's *members* (i.e., traders). As a corporation the organizer is a “complete” organization that decides on the rules of the marketplace (e.g., what is to be traded, how, when and where), and can exert *control* (e.g., that they be traded only on the platform) and impose *sanctions* assisted by algorithmic surveillance (e.g., listing “good” community members on the marketplace homepage, or expelling those online shop‐owners who misbehave), as well as decide on the conditions for gaining and losing *membership* (Ahrne, et al., 2015). The marketplace organizer can also decide what is to be traded on the marketplace and what not. Frequently, marketplace organizers also set up different evaluation devices for what is traded but typically also for controlling the work of sellers and sometimes also the behavior of buyers.

Those trading are in control of their resources and are free to decide whether they will trade or not. Forms of price setting—for example, fixed prices or auctions—are decided by the organization, but concrete prices for whatever is traded are set between the trading partners in a process of mutual adjustment. The marketplace is thus only partially organized (Ahrne & Brunsson, 2011), because the marketplace organizer, even with algorithmic control, does not have access to all the elements characteristic of complete (formal) organization. Hence the organizer creates and orders, for example, an online marketplace (Kirchner & Schüßler, 2019) by making “decisions not only about their own, but also about the behavior and distinctions of others” (Ahrne & Brunsson, 2011, p. 8). Social infrastructure is also affected by the processes of mutual adjustment between actors. As in all marketplaces, cultures are primed by rules, but the local culture—“this is how we do it here”—emerges as an outcome of the interaction of those trading. This emergent order (Berger & Luckmann, 1991), in contrast to a decided order, “merely happens” (Ahrne & Brunsson, 2011, p. 7) as the result of actors adjusting to one another (cf. Berger & Luckmann, 1991). Decisions and mutual adjustment (Aspers, et al., 2020) are likely to coexist and interplay in any empirically observable case, which can also be observed in the case of online marketplaces, to which we now turn.

While the economic sociologists cited above emphasize the trade dimensions of online marketplaces, sociologists of work and labor scholars focus on marketplaces as sites of work. Labor studies and the sociology of work examine working conditions (Kate, 2017; Schor, et al., 2020; Vallas & Schor, 2020), and the manner in which the platform's infrastructure affects working conditions (Curchod, et al., 2020), including digital surveillance of how workers in the food delivery industry experience and resist managerial and algorithmic *control* (Newlands, 2021). Labor process studies have also analyzed platform‐based work, including the process of digitalization, and how the globalization of platforms opens up opportunities also for entrepreneurs from more stigmatized countries (Lehdonvirta, et al., 2018). While this literature informs our study, it is limited in its focus on digital platforms only as work arenas, while neglecting their operation as online marketplaces. In light of this literature eeconomic sociology's notion of “social infrastructure” requires adjustments to analyze online marketplaces as sites of both work and trade. Additionally, it needs to demonstrate increased theoretical and empirical sensitivity to work inside markets and to market work (Bandelj, 2009; Cochoy & Dubuisson‐Quellier, 2013; Darr, 2006).

Cochoy and Dubuisson‐Quellier (2013), for example, show how professionals such as marketing experts, shape not only consumers' tastes, but also their judgment devices. By drawing on funeral services (Trompette, 2005) they show how this work contributes to the commercialization of products and services previously outside the purview of the market economy. Other illustrations of work in markets include “reputation work” (Fine, 2019), the role of occupational communities in shaping market exchange (Darr, 2006), and various types of “consumer work” (Dujarier, 2016), highlighting the unpaid labor of consumers when writing blogs and product reviews and rating sellers (see also Scholz, 2013 for ambarella term “Digital labor” providing a neo‐Marxist perspective on these types of market practices). This literature enriches our theoretical notion of the “social infrastructure” of online marketplaces by highlighting market work related to the creation and maintenance of reputation, the development and application of judgment devices, and the role of unpaid (digital) labor in maintaining the social infrastructure of online marketplaces.

In addition to the abovementioned sociological research streams, the business literature has taken seriously the trading conditions and, in particular, trust in the online economy, arguing that digital trade platforms create “uncertainties for buyers because they mostly transact with new and unknown sellers with no brand name” (Pavlou & Dimoka, 2006, p. 392). Business scholars have addressed the different challenges facing those who organize online markets (Walia, 2013), including how to generate *trust* between buyer and seller (Luca, 2016, p. 77), acknowledging nonetheless that the roots of trust are often to be found outside platforms (Möhlmann & Geissinger, 2018). The social infrastructure in these studies consists, for example, of organized reputation systems attached to the platforms, which seem to be the most frequent approach to signaling who may be trusted (Abrahao, et al., 2017). The possibility of trading using blockchain technology (Parker, et al., 2016, pp. 171–173) to close deals is a unique trust‐enhancing mechanism in online marketplaces, because such transactions are public to, and traceable by, all participants. Obviously, these decided infrastructural systems are subject to manipulation by traders as well as by those organizing the trade (Luca, 2016). Yet another option is to let third‐party agencies perform certifications or verifications of a platform's “trustworthiness” (Cardoso & Martinez, 2019).

The online platform economy's infrastructure is also made up of algorithms and clouds, which open up opportunities for sharing and expanded trade (Kenney & Zysman, 2016), but also create power asymmetries in online markets (Cutolo & Kenney, in press). Indeed, many features of the algorithms and thus the inner workings of the platform remain opaque and inaccessible to regular users. Moreover, machine automated learning of “rules and patterns from data” to draw conclusions without detailed programming (Fourcade & Johns, 2020, p. 804), in addition to more traditional socialization (i.e., “social learning”), should be included in the broader frame of social infrastructure, of which non‐trading platforms, such as Facebook, are also part (Van Dijck, et al., 2018). More generally, terms of trade in online platforms are defined by the organizers of marketplaces, and are often embedded in software.

Table 1 takes a unified theoretical approach, comprising the different elements of the social infrastructure of marketplaces, as presented in the literature we reviewed, analytically divided into those that come about as a decided versus those that come about as emergent order with projected outcomes. The elements are derived theoretically, although some categories are more descriptive. Some elements support the depiction of marketplaces as work arenas, others as trading spaces. Indicative references are included in the table, because it is not possible here to elaborate on the references of all the elements that we use.

**TABLE 1:** Marketplace elements resulting from, and supported by, decided versus emergent social infrastructure

Marketplace element	Social infrastructure resulting from decided order	Social infrastructure resulting from emergent order
Trust in trade (Durkheim [1895] 1984, Luca, 2016)	Can be instituted for example, by rating systems or blockchains	Outcome of buyer–seller repeated interaction (e.g., clientelization)
Membership (Ahrne & Brunsson, 2011)	Decided by organizers	–
Formal rules (Ahrne & Brunsson, 2011)	Decided by organizers	–
Control of rules (also algorithmic control) (Ahrne & Brunsson, 2011; Newlands, 2021)	Done by organizers	Can be done by all actors
Sanctions (Ahrne & Brunsson, 2011)	Decided by organizers	Can be imposed by all actors
Products traded (e.g., Karpik, 2010)	Decided by organizers	Product differentiation by traders (for example, brand names)
Forms of price setting (for example, auction or set prices) (Aspers, 2011)	Decided by organizers	Outcome of buyer–seller interaction
Trading prices (e.g., Velthuis, 2005)	–	Outcome of buyer–seller interaction
Culture of the marketplace (e.g., Beckert, 2010; Wherry, 2012)	Can be conditioned by decided rules	Outcome of buyer–seller interaction
Evaluation devises (e.g., Karpik, 2010)	Decided by organizers (reputation systems)	Status differentiation as traders evaluate one another
Market work (Cochoy & Dubuisson‐Quellier, 2013)	Defined by organizers	Adjusted by actors

The concepts presented in Table 1 will theoretically frame our analysis of our empirical data.

## 3 RESEARCH DESIGN AND METHODS

The empirical study, carried out during 2014–2015, was designed to provide a broad and comparative depiction of buying and selling in online marketplaces. It is based on 54 in‐depth interviews, primarily with buyers and shop‐owners operating in the Israeli platform economy. A snow‐ball sampling technique was used, with an emphasis on multiple foci, to avoid the danger of being caught up in a small number of cliques among interviewees. At the time of the study, the main option for online shop‐owners other than Etsy was eBay, and they could also open an independent shop off a platform and hire online shop designers and promote the shop. Opening a shop on Etsy was considered much easier than on eBay, so once Etsy was established, many craftspeople either migrated to Etsy or also opened a store on the platform. All shop‐owners who were interviewed operated an Etsy shop, and some of them also operated shops on other platforms and independent shops, a fact that allowed them to compare their experiences. Buyers who were interviewed, mainly graduate students in their late 20s and early 30s, had previous experience in buying online, on Etsy as well as other platforms. When we present excerpts of interviews with buyers, we clarify whether the field data pertain to Etsy or to other platforms. The online store designers, online marketing experts, photographers and copywriters, were included in the sample because they provide valuable insights into the network of professionals supporting online buying and selling. They also were helpful in explaining the historical trajectory of this sector, and the unique qualities of Etsy when compared to other platforms. Importantly, a few of the shop‐owners we interviewed used the professional services of marketing and social media experts, and interviews with these actors provided valuable data on their services and the nature of their relationships. Table 2 breaks down interviewees by market‐related role.

**TABLE 2:** Breakdown of interviewees by market role or occupation

Role	No.
Buyer	19
Online shop‐owner	8
Website builder	9
Website designer	9
Publicity and marketing	3
Media manager/programmer	6

In the interviews, attention was paid to market actors' perceptions of the online marketplaces mediating the online exchange of goods and services, and their own personal experiences with online buying and selling, particularly on Etsy. Special attention was also given to the experiences of shop‐owners on Etsy, as well as their ties with the platform, the algorithm running it, and with other shop‐owners. Buyers were also asked to describe different aspects of their relations with shop‐owners, divided into different phases—information search, negotiations and post‐sale communication. The average interview lasted about 40 min. Most interviews were tape recorded and transcribed in full. In a few instances, notes were taken during interviews and later expanded on a computer. The majority of interviews were conducted in Hebrew by two research assistants. Interviews with some individuals of Russian origin were conducted in Russian and then translated into Hebrew by the Russian‐speaking interviewer. The interview excerpts that appear in this article were translated from Hebrew into English by the second author (by alphabetical order).

All transcribed interviews were compiled into a single dataset for coding and analysis. The second author also compiled the questionnaire used in the study, supervised the interviews and conducted the data analysis. One of the shop‐owners on Etsy compiled for this study a log of all transactions she conducted during 1 month, and we had access to anonymized correspondence between market actors related to some of these transactions. She also documented all instances involving gift‐giving as part of transactions. Some of the buyers' comments and ranking of this particular shop were also documented. Additional data sources included a targeted Internet search for statistical data and newspaper articles about Etsy.

The initial analysis of the empirical material was based on grounded theory (e.g., Strauss & Corbin, 1998), which requires interpolation between theoretical concepts and empirical analysis. Data coding began with the construction of broad descriptive categories, such as “attitude toward the mediating platform” or “depictions of interactions within market role and across the market”, and then with sub‐categories, such as “depictions of information search by buyers” and “sellers” requests for favorable ratings from buyers'. More fine‐grained coding then took place within each sub‐category, and hierarchical relations among the categories were established. In the second round of coding, and based on Tavory and Timmermans' (2014) work, the analysis included an “abductive” extension, and some materials were coded using the theoretical notions listed in Table 1. The excerpts from the data presented below are key examples of our findings and help illustrate and clarify the contribution of our unified theoretical framework.

## 4 FINDINGS

The findings demonstrate that the social infrastructure of online trade, encompassing work and trade, is the result of social interactions within a triad of dialectic relationships between the platform, the shop‐owners and the buyers. This process is ongoing and multi‐faceted, but, for analytical reasons, in the following sections we break it up into stages.

### 4.1 Decided order: Etsy's attempts to organize an online marketplace

The socio‐technical affordance of online marketplaces offers their organizers possibilities to design and regulate work and trade. They can inscribe the terms of exchange in algorithms, *control* the structure and flow of communication, control shop‐owners’ behavior, and document and analyze vast amounts of data pertaining to market actors and patterns of exchange. Belief in the ability to design online marketplaces is so entrenched that entrepreneurs sometimes declare their explicit intentions to superimpose tailor‐made order through their algorithms. One of the co‐founders of Etsy provided an illustrative example in 2010. He declared that his goal, when he launched Etsy in 2005, had been to strengthen market communities, so that they can shape economic trajectories rather than be shaped by broader economic changes. Etsy's founders were also explicit about their desire to constitute an online marketplace that replicates a traditional marketplace for crafts. They strove to create tightly knit and active communities of sellers (Pace et al., 2013), and to focus on small and stable enterprises, private craftspeople and artists.

**FIGURE 1:**
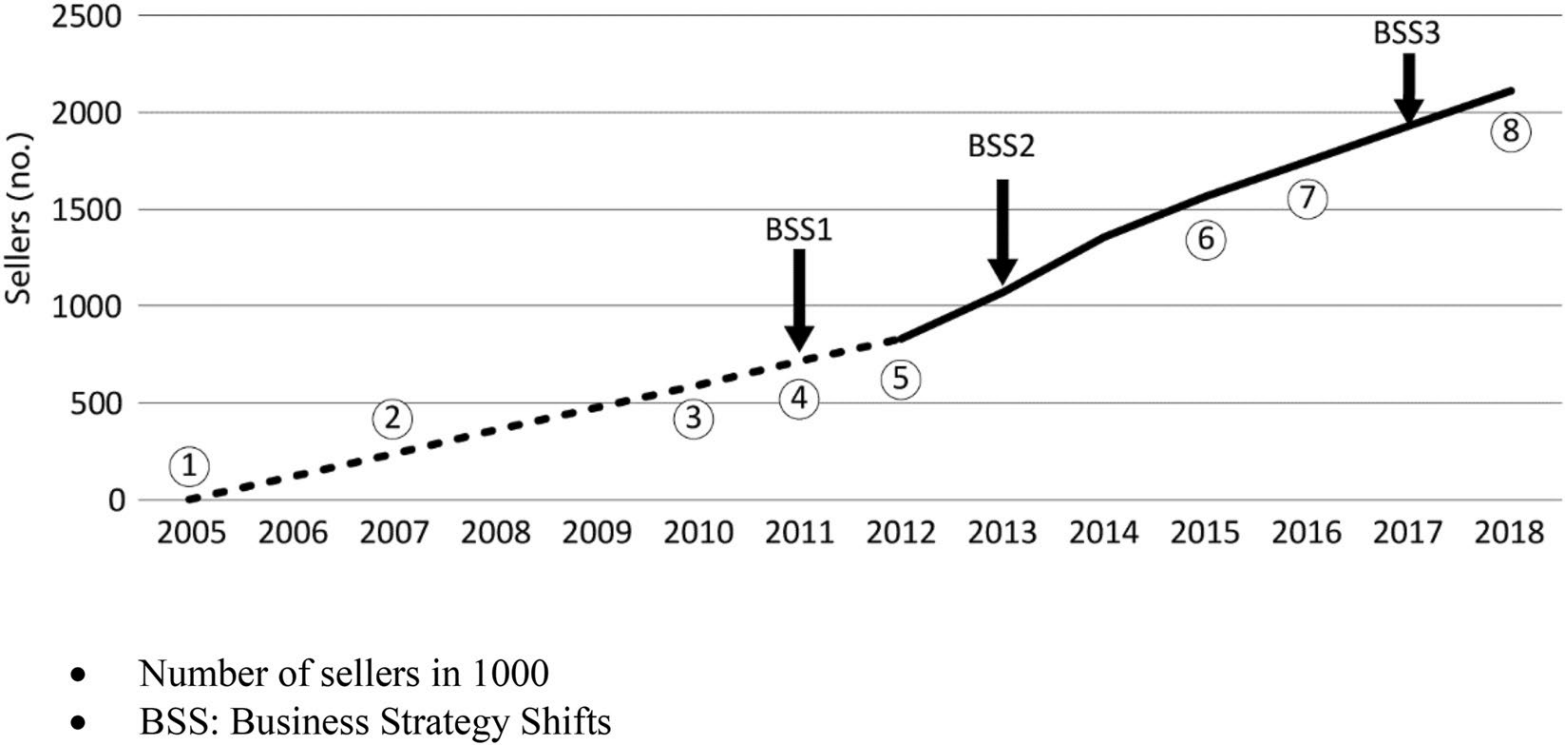
Etsy's lifecycle, number of registered sellers and main business strategy shifts

Etsy's business model at the time of the study was to provide potential shop‐owners with a standard blueprint for setting up and running an online store. It offers a simple way to upload images and information related to items offered for sale. This means that Etsy's user practices are to some extent formatted by Etsy's decisions that are inscribed into the algorithm. Sellers were originally mainly private craftspeople or small businesses producing handmade artifacts and artworks. They continue to operate as business entrepreneurs on Etsy. Since 2005, the year it was established, Etsy's definition of what constitutes handmade items, the *products traded*, craftsmanship and vintage objects, has changed, and what can be offered on the marketplace has changed accordingly. When one opens a shop through Etsy, one becomes a “*member*” of the Etsy “community”, to use the management's vocabulary, and one must follow the platform's rules and its “code of conduct”. Thus, from the outset, Etsy limits the autonomy of shop‐owners who work on the platform. Figure 1 documents the main trajectories in Etsy's lifecycles, the number of registered sellers, as well as the three main strategy shifts that define the ecosystem it has created for its sellers and buyers.^1^

In the following paragraphs we offer brief descriptions and explanations of these key trajectories, organized around the numbers that appear in Figure 1. The three main business strategy shifts (BSS 1, 2 and 3 in Figure 1) are also discussed. We see all these stages as ongoing attempts by the organizers to implement the social infrastructure in the marketplace by making decisions for others. The organizers use contracts, formal rules and normative guidelines for shop‐owners and buyers, and they also inscribe rules and norms in the algorithm that operates the marketplace.

1. Founded in 2005 in Brooklyn, New York, by Rob Kalin, from its inception Etsy has focused on the craft and collectibles section of the economy. Sellers striving to open online stores could choose one of three main categories: handmade items, vintage items or craft tools (Wikipedia.com, accessed December 11, 2019).
2. In 2007 Etsy reported 300,000 deals on its marketplace. With self‐reported steady growth, and a crisis that hit its giant competitor eBay in 2007–2008, Etsy declared that it would strive to create an active community of sellers and buyers to differentiate itself from eBay (Pace, et al., 2013), focusing on small enterprises, private craftspeople and artists. Etsy also claimed that it would verify the authentic sources of products being offered for sale (Krugh, 2014).
3. The year 2011 saw Etsy's first strategic shift (BSS1 in Figure 1), when it introduced a system called “People Search” that allowed sellers and buyers to communicate in the marketplace. The system also included internal feedback and rating, which is a form of evaluation in which buyers could rate and comment on products they purchased and the quality of service of individual shops and shop‐owners. This technological change provided the basis for the future creation of distinct communities of buyers and sellers.
4. In May 2012 Etsy stepped up its efforts to become a major global player in the emerging platform economy by pursuing horizontal integration. It began to purchase other marketplaces that operated online craft markets and to expand the types of products.
5. To attract a larger number of sellers, in 2013 Etsy revised its business strategy (BSS2 in Figure 1) when it declared that it would also allow the sale of machine‐made artifacts in the marketplace, as long as the workshop‐owner is a designer or employs a dedicated designer for the items sold. This strategic shift was also a precursor of Etsy's desire to become a publicly traded company.
6. Etsy went public in 2015 and started to invest heavily in technological innovation, enhancing management's ability to impose a social infrastructure through algorithmic control. A key example is the implementation of Blackbird Technologies, an AI system that improves buyers' search capabilities, offers image searches, and enhances the marketplace's and users' ability to analyze store visits and deals (https://techcrunch.com). At the same time Etsy's founders continued to propagate values such as craftmanship and creativity ‘where creativity is understood as a property of the work process, as a goal for both crafters and Etsy's coders' (Close, 2016, p. 1904). Not long after its share offering, a group of investors launched a class action suit against Etsy, claiming that up to 5% of items sold on the site are suspected of being fake or an infringement of trademark laws. The class action signaled increased scrutiny by Etsy management of the items being offered for sale, and sellers who failed to adhere to Etsy's bylaws could be expelled from the marketplace.
7. In late 2016 and early 2017 Etsy launched its third strategic shift (BSS3 in Figure 1), deeply rooted in algorithmic technologies, when it introduced a peer2peer system that allows sellers to communicate among themselves and to promote social ties and a sense of community. Additionally, the peer2peer system enhanced the rating system and allowed buyers to respond more easily to other buyers' reviews and to communicate with one another through this system. Importantly, peer2peer systems are perceived by buyers and sellers as fair and just (Pazaitis, et al., 2017), and they encourage collaboration among *members*. They encourage sellers and the formation of virtual entrepreneurship that emerge out of this cooperation, as well as the solidification of buyers' shared interests.
8. At the time of the study, Etsy earned income by charging its members—those selling items in this marketplace—a fixed fee, amounting at the time of the study to US$ 0.20 per item offered for sale, and also by charging a 3.5% commission on each sale made through the site. Store owners, who, according to statistical data on over 14,000 French shop‐owners on Etsy (Jourdain & Naulin, 2020), are mostly women (88%), are also required by Etsy to pay a fixed monthly fee for the promotion of their wares. Marketplace organizers make a profit from the large volume of trade, so they aim to attract many high‐frequency traders. Based on this historical background of Etsy's purposeful attempts to constitute an online marketplace, we shall now examine the following question using our own empirical material: what is the experience of sellers who work with Etsy?

### 4.2 The experience of sellers with the decided marketplace order

Dalit, a 36 year‐old craftsperson who operates four online stores in different Internet marketplaces, describes the standard features Etsy offers to prospective virtual store owners. Dalit highlighted the tight *control* of Etsy's algorithm over shop‐owners, and says that Etsy associates cannot change the site's format for presenting the items they sell; they may only change “the order of presentation of items, or, for example, if I want the first page [*of my “shop”*] to be all white”.^2^ She elaborates on her example: “Let's say I want that on the first page only the white items will appear, on the second all the pink products, on the third all the blues. And that is it!” Although Etsy offers limited options, it attracts many potential store owners who have little or no technical ability or start‐up money to hire professional designers. But the marketplace's organizers offer *members* more than a simple format for online shops. Etsy also promotes a sense of what it calls “community” among store owners.

One interviewee who was asked how she promotes her virtual shop, replied, “I pay a sum of money each month for promotion within the site itself, within Etsy. I also do some kind of work … this is a type of community work I perform inside Etsy, on a daily basis. I took a class to understand how it works”. In the following quote this shop‐owner expands on what she means by “community work”.

… Etsy has a ‘front page’ [*said in English, meaning the home page*]. Each time, a different craftsperson [*i.e., an Etsy member*] compiles a collection of works of about nine other craftspeople from the site, and Etsy’s crew chooses every hour a different collection of works [*for the Etsy home page*]. So actually, it turns out that if I prepare these sheets [*collections*] for others, each of them will prepare one for me, and this advances my chances of reaching the front page.

***Interviewer:*** And what does it depend on, you reaching the front page?

***Shop‐owner:*** Truth is, it depends on lots of things: my photos; if I include other people on my sheet.

***Interviewer:*** So this means that you promote other people on ‘your’ Etsy site?

***Shop‐owner:*** Yes…. I help other people to move forward, and they help me. This is the foundation [*of the community*].

This long excerpt reveals how Etsy has regulated *reputation work* on the platform, and how it organized its marketplace around a code of conduct, *rules* that motivate its member shop‐owners to collaborate rather than merely compete, to form an online community of sellers who help each other promote their products, and the platform. The marketplace even offers training to shop‐owners on how to foster cooperation. Importantly, economic sociology has emphasized evaluations and judgment devices (Karpik, 2010), as well as traders' reputations (Podolny, 2005) long before more recent studies of online marketplaces. However, algorithmic control fosters greater regulation of these elements of social infrastructure.

The same interviewee explained the positive *sanctions* after one's collection of members' work, dubbed “treasure” by management, appears on Etsy's front page: “When clients open the front page, you have more exposure since your name appears on top, as the one who put the collection together, with the names of the other people [*whose work you chose to appear*]”. Etsy's management directs some of the work efforts of shop‐owners to promote the platform and its ideology. It encourages active participation of groups of shop‐owners to organize and serve as “tastemakers”, who do a type of market work that should be viewed as unpaid “*digital labor*”. Together with Etsy staff, members of these groups choose collections of photos of their products prepared by group members, and these collections appear on Etsy's front page in hourly rotation, which is a positive *sanction*. Thus, the ability to advertise their products collectively has served Etsy organizers as an important motivation device to create bonds among the shop‐owners.

The algorithm is employed by Etsy *monitors* shop‐owners, and is designed to collect information about the degree to which they participate in the collective creation of “treasures” and in different groups of “tastemakers”. The ratings of shop‐owners on the basis of their “community work” affect the chances of their products appearing on the front page. It should be seen as part of the algorithmic control of shop‐owners, and as a form of *ranking* sellers.

Some shop‐owners fail to cooperate with Etsy's decided order, and economic failure is often the result. The owner of one failed Etsy store claimed that online selling through this online marketplace requires what can be dubbed “*market work*”, the constant care, community and promotional work:

My business is jewellery design… I started with Etsy but I didn’t do very well, because there, you need to constantly do something [*for Etsy*], and I didn’t have the strength or the will… This is how you really need to do it every day, for a few hours, to dedicate to Etsy. I mean, people who really succeed there, they do it. They make pictures [*i.e., ‘treasure’*], you have a special aesthetic of photos, which… attract more attention. I mean, you really must sit down and work with this site.

Some level of choice and social agency is retained by shop‐owners because they can refrain from creating ‘treasures’ or becoming “tastemakers”. According to this account, however, economic failure is likely to result. *Market devices* implemented by the algorithm provide shop‐owners with opportunities to engage in “*reputation work*” in several ways—for example, by encouraging buyers to recommend their stores to others. The shop owner cited above explained, ‘You can ask people, friends [*playing the role of “buyers”*]: “Please click on my treasure,” since when you collect more clicks, your ratings go up’. Some reputation work is carried out off the platform, through social media and independent marketing experts.

As Table 1 indicates, decided elements of social infrastructure in traditional as well as online marketplaces are supported by sanctions. Etsy is a site of work for the shop‐owners, not only a trading arena. The more formal, or contractual, and hierarchical relations that exist between Etsy's management and shop‐owners are reflected in the sanctions it imposes on sellers who do not behave as is expected of them. A few interviewees claimed that the formal sanctions they experienced made them leave Etsy in the past, only to return at a later stage to this central platform in their field. Sanctions are typically the consequences of buyers' ratings, which are viewable by anyone trading on the platform. These ratings significantly affect the seller's success or failure, and sometime form the basis for formal complaints. A shop‐owner explained:

Etsy has a feedback system in place, so the customer knows that if he [*sic*] fails to receive the merchandise, doesn’t get what he ordered, he can complain to Etsy and Etsy will protect him [*sic*]. They can launch a complaint and even get to the stage where they close your store. This is Etsy.

Through customer feedback, a built‐in affordance of the algorithm, Etsy attracts buyers by providing them with a layer of protection against fraud. Not only can buyers share their purchasing experiences with other buyers, but they can also complain to Etsy about unsatisfactory sellers. Sellers cannot avoid this *control* mechanism, but can complain when they feel that an unjustified decision was made in favor of the buyers.

Etsy offers buyers and sellers an arbitration process to help resolve disputes. When buyers feel sellers are misbehaving, they can enforce arbitration by launching a formal complaint. When shop‐owners fail to adhere to the results of the arbitration process, they risk being expelled from the marketplace. In their interviews, a few sellers expressed the view that Etsy favors buyers, an argument that has also appeared in the press. Here, the intersection of work and trade is particularly clear.

A veteran seller on Etsy, who also owns other virtual shops, among them independent stores operating outside platforms, argued that sellers are at a disadvantage vis‐à‐vis buyers because, as she put it: “When you have a store on Etsy, you have someone higher up [*the platform organizers*] who actually represents the buyers. In independent online stores you don't have that”. This speaker added that sellers on Etsy tried to promote a rating system for clients, to weed out buyers who systematically demand refunds or commit what she called “feedback violations”, for example, writing negative feedback without contacting the seller first. According to this account, this initiative was first accepted and implemented by Etsy, but only for a very short period. This is but another example of sellers' limited possibility to affect the infrastructure that conditions the trade.

As a result of this centralization and technological affordance, virtual marketplaces have very “visible hands”. The organizing companies shape marketplaces for others, primarily sellers but also buyers. They make the decisions, set *rules*, *control* participants, impose *sanctions*, both positive and negative, and extract unpaid “*digital labor*”, to steer the activities and behaviors of *members*.

### 4.3 Buyers' experience with the marketplace's decided order

Table 1 postulates that constituting trust is a central element of social infrastructure in all marketplaces. The challenge is exacerbated in online trade, because shoppers pay before they receive their goods. Online buyers in particular are often required to take a leap of faith (Möllering, 2006). In online marketplaces the social infrastructure needs to compensate for the fact that traders, though virtually close, are physically distant and therefore unable to touch and closely examine the merchandize, and may operate within different institutional and legal frameworks. Online marketplaces may, much like the fortified marketplaces in the Middle Ages, provide security for traders and promote *trust*. Relying on the reputation of a marketplace's brand name (Podolny, 2005) and safety mechanisms inscribed in its software, such as rating systems, buyers can take this leap of faith. Informants in a few interviews highlighted the role of offline friends in constituting trust in specific online marketplaces, such as Etsy. Drawing on this evidence, we suggest that existing offline ties with friends and peers are integral to the social infrastructure of Etsy, as well as other online marketplaces.

Etsy should be viewed as a fixed‐role marketplace (Aspers, 2011), that is, a marketplace in which actors gain an identity as either seller or buyer and share the interests of others in a structurally equivalent position. Buyers can communicate through social media, but these virtual communities coalesce mostly around rating systems embedded in trade platforms, where members, by performing unpaid “*digital labor*” collaborate to assess the quality of goods offered for sale and the trustworthiness of different sellers. To be sure, marketplace organizers use different means, including rating and other *evaluation devices*, to facilitate buyers' judgments of product quality. They may restrict the number of sellers of a type of product (Pradelle, 2006), categorize goods in different ways (Hsu, et al., 2009), and establish standards and tools for evaluating quality (Podolny & Hsu, 2003). Rating of sellers by buyers is another, central, means of constituting trust and overcoming the problem of trading unique products (Karpik, 2010). The rating systems go beyond the intentions of marketplace organizers, and are analyzed below as in part an unintended consequence of their desire to design and control rating systems. A 28 year‐old graduate student highlights the central role of rating systems, on Etsy but also on other platforms, for establishing a sense of a community of buyers.

On the internet each product has users' reactions attached to it, and you can find products with thousands of buyers' reactions. And you can go through them. People who purchased the item, what do they say? Including product malfunctions, repair, insurance—everything.

Underpinning the motivation of buyers to engage actively in unpaid labor in writing product reviews and ratings is their ability swiftly sanction faulty sellers, a feature lacking in bricks‐and‐mortar stores. A search of buyers' comments on Etsy, which focused on a particular online shop whose owner was interviewed for this study, revealed a few negative, but mostly positive and even personal pieces of feedback. A typical comment, accompanied by a picture depicting an infant in his crib, read: “Our little rock star is finally old enough to appreciate your pillow so I thought I would share. Stay creative!” Through the rating systems, online buyers constantly observe the reactions of other buyers and collectively lower their market uncertainty and further their shared interests.

Rating systems are part of our notion of social infrastructure. They show *horizontal ties* between the marketplace organizer and the shop‐owners. Ratings systems are decided by Etsy to coerce shop‐owners to react swiftly to clients' complaints or poor ratings and to avoid arbitration and sanctions. Viewing platforms as work sites illuminates how buyers are turned into rule enforcers and part of the control of shop‐owners, a practice similar to the deployment of fake customers as monitors of individual employees in the service industry (Fuller & Vicki, 1991). Our treatment of rating systems complements previous studies that have analyzed them as trust‐building mechanisms and as a means of producing certified knowledge—for example, in the form of standards (e.g., Banks, 1963). Rating systems trigger the creation of social ties within and across market roles, and in many respects substitute for prices (Karpik, 2010) as main market signals. While rating systems can be seen as a form of communication between sellers and buyers (Esposito & Stark, 2020), we argue that they represent a form of *market work* by buyers, in this case because they function primarily to enhance and display the similar interests of the online community of buyers. Importantly, as compared to their tight control of shop‐owners, Etsy cannot control the communication among buyers through the rating systems. Etsy can delete unfavorable ratings of the shops, but such actions are rare, according to some of the shop‐owners, and might result in public shaming through social media and the migration of buyers to other marketplaces.

Our interviews support the claim that in online trade the seller's rating has replaced product price as the main market signal affecting buyers' purchasing decisions. A 31 year‐old engineer focused on the role of ratings as he described more generally the main differences between online and offline trade:

I think that most salespeople in a [*regular*] store, they want to sell, so they also lie and cheat, and they do that so I’ll purchase an item and pay money. This is also true for the internet, but on the internet… if a seller sold me something and I’m not satisfied, if the description of the seller is inaccurate, and if, while communicating with me in writing, the seller wasn’t sufficiently polite and didn’t react reasonably, I can give him a shitty rating, something which hurts his reputation and the reputation of the specific site. And then other users, other people are more aware. This is different than going to the shopping mall and, let’s say, receiving poor service. So what do I do with that? What? I’ll go to Facebook and start a group: ‘Listen, I went here and got that.’ Who will be interested? Here [*online trade platform*] I enter and write ‘Shitty service. What I ordered wasn’t similar to the photo… give me my money back!’ Just like that. And returning the money is nothing [*for sellers*]. What is real is that I hurt his reliability, I mean, serious sellers who have a good name are worried [*about losing it*].

This buyer points to the role of the rating system in creating a community of buyers, a grassroots creation involving seller–buyer interaction along the platform's technological affordance.

In addition to marketplace‐instituted rating systems, a variety of other online judgment devices (Karpik, 2010) exist, including reports, blogs and third‐party rating systems. All of these devices exist on and surrounding Etsy. Social media is used extensively by shop‐owners to advertise their shops and to consult and communicate among themselves and with prospective buyers (Blanchflower & Hodges, 2015). Buyers on Etsy reported that they write blogs and communicated with shop‐owners on Facebook in dedicated groups. All these activities should be seen as a type of *market work*, that contributes to an emergent online marketplace *culture*. More generally, digital marketplaces such as Etsy and other Internet fora enable consumers to band together, essentially constituting a social movement (Fligstein & McAdam, 2012) with an organized voice that affects individual market operators.

### 4.4 Social infrastructure resulting from mutual adjustment

Interactions between sellers and buyers on Etsy are often structured around the rating system with its distinct technological affordances. What evolves from these interactional data is a form of emergent order, in which sellers and buyers trade with the resources that the decided order provides. In the following example, buyers' ability to provide feedback is transformed from an objective evaluation of sellers to a bargaining chip in the ongoing negotiations between buyers and sellers. A buyer on Etsy described how he was able to receive a refund without sending the item he purchased back to the shop‐owner:

When the item I purchase costs only a few dollars to the seller, so he might simply say, only to avoid a negative feedback that I might write, so he [*the seller, sic*] says: ‘I’ll return your money. Don’t send the item back to me’. … In other cases the seller wants the item back, but it only cost me two dollars, and mailing it back will cost me more. So it is not worthwhile for me. So I explain the situation to the seller, and I tell him, ‘if you don’t want to return my money [*without receiving the item back*] I’ll simply write a negative feedback’. And in most cases it works.

Shop‐owners on Etsy can ask to receive their items back before issuing a refund, but in this example the buyer threatens negative feedback to avoid returning the purchased items. This clearly exemplifies how buyers sometimes use their ability to write negative feedback afforded by the platform, as a bargaining chip in creative ways that go beyond the marketplace organizer's original intentions.

The rating system on Etsy also underpins a thriving practice of gift‐giving in this online marketplace. Gift‐giving solidifies social ties and creates social obligations to reciprocate (Darr, 2006). Shop‐owners systematically use gifts to try to secure favorable ratings from buyers. The amount of gifting on Etsy is surprising. The log of all transactions during 1 month compiled by one Etsy shop‐owner reveals that a gift, a small hand‐made cloth bag, was sent with each and every shipment. Returning buyers or those making large orders received additional gifts. Reciprocation took many forms, one of them a tacit or more explicit expectation from clients to write positive reviews. Thus, we suggest that gifting should be viewed as an example of shop‐owners’ agency, a type of *market work* designed to solicit and even manipulate the technology‐enabled ability of buyers to write reviews that are shared with all other interested buyers.

Finally, shop‐owners’ ability to collaborate, which is offered by Etsy, and the incentives the platform provides for doing so, has some unintended consequences. For example, one interviewee reported how previous offline ties among shop‐owners shape forms of collaboration. She claimed that offline friends who open shops on Etsy tend to cooperate repeatedly and to create cliques, rather than constantly change partners, as they work together to create “treasures” and to further their mutual goals. This is an indication that offline and online marketplaces are always interconnected. More importantly, this tendency to rely on offline acquaintances when collaborating on Esty should be viewed as yet another element of the emergent social infrastructure of the online marketplace. Here, as in previous examples, the marketplace provides the prescribed behavioral scripts, and actors improvize and transform them through social interactions.

## 5 DISCUSSION AND CONCLUSIONS

This article offers a novel theoretical framework that systematically integrates two central dimensions of online marketplaces, work and trade, into a comprehensive theoretical scheme. Our approach is designed to remedy existing studies' tendency to analyze platforms as either sites of work or as trading arenas. We argue that the theoretical notion of “social infrastructure” is particularly apt to capture the unique intersection of work and trade that online marketplaces provide. We further contribute to the literature by demonstrating how the social infrastructure on Etsy, which enables and controls online work and trade, is constituted. Social infrastructure results from a combination of purposeful attempts by marketplace organizers and a social process involving interaction and mutual adjustment that create and maintain a grown order.

The decided elements that we empirically identify are made up above all of rules, and the control and sanctioning of members, most of which is directed to the shop‐owners working on the platform. Unlike offline marketplaces, the platform's technological affordance allows much closer surveillance and measurement of market behavior, and the implementation of algorithmic control, of both trade and work, is more intrusive than traditional means of control in the offline marketplace. Those who trade through Etsy must become members of the marketplace and formally accept its rules, and those who fail to do so might be expelled. Shop‐owners are also required to perform unpaid reputation work for themselves and for the platform. Hence, a clear hierarchical, work‐related aspect of trade emerges. However, shop‐owners and buyers exercise agency as they trade and mutually adjust, and when they strive to improve their market position. They are not obligated to maintain an exclusive relationship with a specific marketplace but are free to operate shops and to trade on additional trade platforms. Thus, the Etsy case‐study not only demonstrates a general structuration principle of marketplaces' social infrastructure, but also reveals how the intersection of work and trade is key for analyzing the formation of social infrastructure of online marketplaces.

We find that rating systems comprise a pillar of the social infrastructure of online marketplaces. The prominence of rating systems derives directly from their ability to utilize hierarchical work relations and algorithmic control of shop‐owners, and trade ties across market roles and within communities of buyers that coalesce around them. The intersection of work and trade underpins the creative use of resources by some buyers, who employ their ability to provide negative feedback or to complain about shop‐owners as a bargaining chip to extract economic benefits from sellers. Here, hierarchical ties between the platform and the shop‐owners are utilized by buyers to improve their trade conditions. The ability of Etsy to exercise hierarchical authority as it controls, directs, rates and sanctions the activities of shop‐owners looms large when shop‐owners engage in “community work”. Etsy employs hierarchical relations to encourage shop‐owners to perform unpaid “community work” that should be seen as a specific form of market work (Cochoy and Dubuisson, 2013), monitored by the algorithm. High ratings on active participation result in improved trading conditions for shop‐owners, when their products are displayed on the platform's front page. Community work should also be conceived as “reputation work” (Fine, 2019), by and for the members of the group of shop owners. Thus, work and trade dimensions are entwined in the daily operation of online marketplaces, and the application of our extended notion of “social infrastructure” can provide an essential theoretical framework to capture both dimensions and their important interrelation.

Our theoretical framework, formally presented in Table 1, provides future studies with an integrative analytic tool for the study of online marketplaces. Our finding also open‐up new research venues. Future research can compare and contrast different types of entwining of trade and work elements in algorithms that govern different types of online marketplaces, to better understand how traders and workers are affected by online marketplaces. In such studies, which we envision being conducted as a large‐scale comparative endeavor, attention should be given to the interrelations of decided and grown order. In a similar vein, a highly contested question among scholars as well as in public discourse is the degree to which small entrepreneurs and traders can enjoy ‘market freedom’ on online marketplaces. Empirical studies of online marketplaces, employing our notion of social infrastructure, can provide empirically grounded answers to this central social issue.

## Data Availability

Research data will not be shared.

## ENDNOTES

^1^
Up to 2012 these data are questionable, being based on Etsy's self‐reporting, and reflect steady linear growth (see the dotted line). Since 2012 more reliable sources have started to publish data on the number of sellers on the marketplace that Etsy has organized (https://www.statista.com/statistics/409374/etsy‐active‐sellers/). These data are verified by a number of sources.

^2^
All quotes are verbatim, subject to translation into English.
